# The Surgical Treatment of Dysmenorrhœa*Read before the Medical Society of the County of Albany, N. Y., March 5th, 1876.

**Published:** 1876-07

**Authors:** O. H. E. Clarke

**Affiliations:** Cohoes, N. Y.


BUFFALO

### Medical and Surgical Journal.

VOL XV. JULY, 1876. No. 12.

### Original Communications.

:o:

ART. I.—*The Surgical Treatment of Dysmenorrhœa.** By O. H. E. Clarke, M. D., Cohoes, N. Y.

In presenting this subject it is necessary to consider the following enquiries:

1st. Is Dysmenorrhœa a Surgical Disease, and amenable to Surgical Treatment?

2d. Which are the cases in which benefit may be expected from treatment?

3d. What Surgical means is the most available, and promises the most certain results?

Many, but a few years ago, would, in undertaking to treat a case of Dysmenorrhœa, commence with feelings of misgiving, and would remark to the patient that she must have patience, that many suffered as she did, that if she were married it would benefit her, &c. The unfortunate patient would no doubt be willing to marry could that be reasonably accomplished, but circumstances often forbade even that doubtful remedy. The disconsolate maiden with no prospect of relief often gave herself up to melancholy, or to gin and opium.

* Read before the Medical Society of the County of Albany, N. Y., March 5th, 1876.

Many, no doubt, like myself, have passed this period of transition, and now meet these cases with courage and confidence.

What has caused this change? for myself I can say, the conviction that Dysmenorrhœa depends generally on some mechanical obstruction to the flow of the menstrual fluid, that cases are due to other causes I do not doubt, but the proportion yet remains unsettled, but as our means of diagnosis become more perfect the number of cases dwindle; which cannot be explained.

Dr. J. Marion Sims, in his uterine surgery, gives a list of 129 cases, which he divides into 100 cases of painful and 29 cases of excessively painful menstruation. In the first category the os uteri was normal, but in six cases in the second none were found normal, while contraction or flexion were present in more than 90 per cent. of all the cases.

So convinced is this author of the mechanical causation of this malady, that he lays it down as an axiom, that there can be no Dysmenorrhœa if the canal of the neck of the womb be straight and large enough to permit the free passage of the menstrual blood.

Dr. Graily Hewitt in the last edition of his work, says: "Starting with no preconceived theory on the subject of uterine pathology, it has been my object since I have been sufficiently informed and experienced in various methods of investigation, to endeavor to associate the sufferings of the patient with the normal condition giving rise to it.

The conclusion has forced itself on me that the changes in shape and position of the uterus, but especially in the shape of the organ are almost responsible in one way or another for the suffering of the patients, who are the subjects of them, and further the conclusion, no less inevitable, that the restoration of the proper shape of the uterus is the means of removing these sufferings."

I hold it as unquestionable, continues Hewitt, that Dysmenorrhœa is to be regarded as a symptom indicating in almost every instance an impediment to the escape of menstrual fluid from the uterus.

In support of his position he gives statistics of his large experience at the University College Hospital, London. Out of 624 cases of severe uterine disease which were carefully examined, 377 were found to be due to material change in the shape and position of the uterus, there being 296 cases of flexion and 81 of prolapsus.

All of Hewitt's cases of Dysmenorrhœa excepting seven (most of which were imperfectly examined,) were referred to flexions or prolapsus.

These authors represent what has been called a bias in Gynecological Science, and the facts from which their deductions are drawn, as well as those conclusions themselves, have been considered by high authorities as based on a misconception of the true anatomy and physiology of the uterus.

The real starting point in uterine pathology according to Graily Hewitt, is flexion—a change of shape of the uterus, whereby partial strangulation of its tissues is produced, resulting in chronic congestion of the body and fundus of that organ. Among the consequences of that condition, is an increase of secretion from the lining membrane of the uterus. This being pent up by the flexion, it produces in conjunction with the other effects of chronic congestion, that train of symptoms which have been regarded diagnostic of Endo-Metritis.

The opposite school, following the original views of Dr. Henry Bennett, believe inflammation of the lining membrane of the cervix to be *the* great uterine disease, while Scanzoni has shown that the whole of the uterus must be included in most cases.

A striking example of the manner in which the *same* fact will be variously interpreted by different minds is afforded by the results of treatment in these cases. While the disciples of Bennett and Scanzoni believe that the application of caustics &c., to the uterine mucous membrane acts beneficially by curing the inflammation present, Hewitt holds that it is the dilatation and consequent straightening of that organ which necessarily accompanies such treatment, to which most if not all the benefit obtained must be attributed. The whole of this is denied emphatically by Dr. Henry Bennett, in answer to a paper on the subject by Dr. Matthews Duncan, alike as to its anatomy, physiology and pathology, and he holds up to ridicule the whole as "*imperfect surgical notions*," which are often substituted for what he calls "*rational treatment*," while Dr. Wm. Cummings of Edinburgh, borrowing the very thunder of his opponents, declares that when the bilateral operation is performed, it is the *hemorrhage alone* that does good, and he proposes to substitute scarifications for all such proceedings. I believe this writer uses a very appropriate phrase when he says he *assumes* that stricture is very rare, and exaggerates somewhat when he characterizes the treatment of those who believe in the theory of obstruction as "heroic modes, the details of which are sufficient to make one's hair stand on end!

In a lecture delivered in the Bellevue Hospital Medical College, and published in the *New York Medical Record*, in January of this year, Dr. E. R. Peaslee, after reviewing the various theories that have successively reigned in Uterine Pathology since 1835, terminates by maintaining that it is the oversight of congestion as the starting point of most uterine disease and its being mistaken for inflammation that has lead to so much confusion.

Thus do opinions in our profession, as in all human affairs, tend to extremes, but the very enthusiasm with which each inquirer investigates in the interest of his own theory, increases the amount of general information, and promotes the final cause of scientific truth.

Avoiding the discussion of these views as beyond the scope of this paper, and confining ourselves to dysmenorrhœa, let me venture to support the opinion that as far as this disease is concerned we should adopt the views of Graily Hewitt, Marion Sims, Savage, Greenhalgh, and their followers, that this complaint in a vast majority of cases is due to some mechanical obstruction to the flow of the menstrual fluid; and since my attention has been called to the investigation of such cases, I further believe, as does Hewitt, though perhaps not to the same extent as that author, that in most cases, *flexion of the uterus* will be found at the root of the evil, and that its remedy will relieve the disease.

Dr. Matthews Duncan, who is in direct opposition to Dr. Henry Bennett, with respect to the normal condition of patency of the internal and external os uteri, nevertheless does *not* believe that contraction is the true cause of dysmenorrhœa, but attributes the majority of cases to what might be termed *idiopathic spasm.* Yet Dr. Duncan has found that dilatation with sounds until a No. 16 can be passed, is the best means of affording relief. Four years ago Sim's Uterine Surgery caused me to take a special interest in these complaints, and since that time, I have kept short notes of every case of confirmed dysmenorrhœa I have treated. I find them to be fifty-eight in number, and will give a table of the diagnoses which formed the basis of treatment.

In the fifty-eight cases there were found,

| | |
|---|---|
| Anteflexion of the uterus, (sometimes combined with version,) . . . . . . . . . . | in 20 |
| Antero-lateral-flexion, (in one of which there was version,) | in 3 |
| Retroflexion (sometimes combined with version,) . . | in 12 |
| Small cervix uteri with narrow cervical canal, . . . | in 12 |
| Enlarged or engorged cervix, apparently from inflammation—no flexion being present, . . . . . . | in 4 |
| Polypus, . . . . . . . . . . | in 3 |
| Tortuous cervical canal, or curved cervix, . . . | in 2 |
| Cases referred to Neuralgia, (in which there was no discoverable cause,) . . . . . . . . | in 2 |
| Total, . . . . . . | 58 |

The results of treatment in these fifty-eight cases were as follows:

All received some benefit, and forty-seven were much pleased with the great change for the better that followed.

Of the eleven who were but little benefitted, not one persevered two months in the treatment, and have been lost sight of. Of the forty-seven who were much benefitted, twenty-one were relieved so permanently and effectually that they considered themselves cured. Sixteen ceased attendance as soon as considerably relieved, and have not been heard from since, while three are yet under treatment. The seven remaining although better than before treatment cannot be called cured—but as almost all these work every day, and had to do so while the treatment was being pursued, this circumstance must be received in attenuation of the number that did not obtain lasting relief. I have excluded altogether from this list those who after a few visits ceased all treatment.

I believe that these results, under the circumstances, justify the assumption that the diagnosis was in most cases accurate, and shall therefore maintain the opinion, that by far the most important, because the most frequent cause of dysmenorrhœa is flexion of the uterus, and that next in frequency is tenuity of the canal of the cervix uteri.

Assuming that you have granted the question, I trust you will excuse a rapid glance at some of the causes and effects of flexion of the uterus.

The womb has for its special object the propagation of the species, and in order to do so, it must expand to an enormous extent, to form a temporary home, for the frail creature which is one day to become a man or woman. Were it attached to surrounding tissues, in all its parts, this would be impossible; and we accordingly see, that although it is firmly united with the bladder and rectum, in its cervical portion, the body of the uterus is comparatively free. It is true, we have the broad ligaments attached to the sides of the uterus and parietes of the pelvis, which, to a certain extent, restrain its motion laterally, and the round ligaments, which prevent its falling backwards, in a much more imperfect manner; but there is no special provision against anteflexion, unless it be the presence of the bladder, which of course is not always full.

What then, it may be asked, generally keeps the uterus upright? the answer is, the *thickness* and *resistance* of its own walls.

Many physicians figure the uterus to their own minds as being a hollow viscus. This is a great mistake; they should always think of it as a *solid body*, having in its center an extremely small cavity, the walls of which are almost in contact. This solid fleshy body is firmly held in its place below by the vesico-uterine and recto-uterine ligaments, connective tissue and blood-vessels, and holds itself upright, in the normal state, mainly by *its own* stiffness and solidity.

We will now imagine the case of one of our mill-hands, so frequent in Cohoes.

Our patient has to work many hours in the day, in a position bent forward, running a sewing-machine or pursuing some similar employment. Her labor and confinement are almost constant, her living possibly not of the best, and she becomes anemic and sickly, which cause her tissues to become less well nourished,—more soft and yielding. But her labor continues, and her constrained position for many hours a day is still the same. As a consequence, the uterus, which, as we have seen, is attached below but free above, become gradually doubled on itself in an anteflexed position. This anteflexion, constricting the vessels of the uterus which almost wholly pass along the upper part of the cervix, causes a certain amount of strangulation of the uterine tissues, with chronic congestion of the fundus and body and its results.

Among these consequences, one of the most frequent and remarkable is dysmenorrhœa.

The flexed, and therefore constricted cervix, offers a smaller channel than before to the exit of the menstrual fluid, while the chronic congestion in which the organ is maintained, is likely to increase its amount. Both these circumstances combined, the lessened channel of exit, and increased material to pass through it, causes *dilatation* of the cavity of the uterus, which now retains a certain amount of blood; this, in its turn, provokes forcible action of the uterine walls, to expel the contents, which continuing, brings on *hypertrophy of the uterus* with still more *congestion, dilatation* and *flexure.* Thus when once a certain point is reached, matters go from bad to worse if not relieved. The pleasures and exertion, combined with the irregular and imprudent diet, incident to a career in which personal enjoyment is the great aim of life, place our wealthy ladies in as much danger of contracting these maladies, as their humble and less fortunate sisters.

I have chosen but one cause of flexion as an example, leaving to your own well informed sagacity, to suggest the others, but I cannot refrain mentioning imprudences at an early date after parturition,—when the uterus is soft and large—as a common starting point for these ailments; nor can I fail to indicate sterility and abortion, as among their frequent consequences.

In support of the fact that flexion *alone*—without narrowing of the cervical canal, properly so-called, or inflammation, is a cause of dysmenorrhœa. I will cite a few out of several cases.

Miss K——, aged 27, called on me last month to consult about a pain in the right side, which was almost constant, and increased by exertion. This pain had been present for two or three years, and for about the last year her monthly periods were accompanied with much distress, which became more and more severe. The patient works and oversees in a knitting mill, and is either on her feet or sitting down and bent forward during her working hours.

On examining I found the uterus to be very much anteflexed. Her monthly period being about to occur, I explained that I would make no examination with the sound, nor commence treatment, until after she had menstruated, except by placing a proper pessary, to support the uterus in a better position. She returned after her period was over, and said she had not menstruated with so much ease for a long time, and that the pain in her side was very much relieved. I now passed a No. 10 male sound, with great ease, by pushing the fundus backwards with the hand on the abdomen. It entered fully two and a half inches,—the patient is a tall, well developed young woman.

Now, in this case it is evident that there was no real *narrowness* of the cervical canal, properly so-called, but only induced *narrowing* from flexion—neither was there any inflammation, the uterus bearing manipulation with little pain, finally the relief attending the adjustment of a proper support showed both the nature of the case and the treatment indicated.

The following case which applied since completing the list I read, shows the same with respect to retroversion.

Miss E. R., sent for me about six months ago for an ishio-rectal abscess which I opened. She then told me she was subject to much straining, burning and itching about the anus, which prevented her sleeping at night, and she was very costive. Prescribed a saline purgative and anodyne lotion, which gave some relief. Told her she had probably retroversion, and proposed an examination which was declined. A few days ago she came to the office with same complaint. Says she also has frequent desire to micturate, which causes pain. Agreed to an examination, when I found the uterus completely retroflexed and retroverted and lying fairly across and low down in the pelvis. By giving it the proper curve, I could readily pass a No. 8 sound three inches backwards into the uterus, showing that organ was enlarged, but that there existed no real stricture of the tube, and as the uterus was unusually tolerant of interference, it proved no inflammation was present. Yet this patient besides the symptoms already given, is so sick when menstruating as to be obliged to leave her work and go to bed, and her agony of severe remittent pains continues increasing until on the third day she vomits forcibly and thereby expels a certain amount of clotted blood, after which she begins to get better.

I replaced the uterus and applied a pessary—gave no medicine. Patient called the next evening to say she had not passed so agreeable a day and night for years, and that the burning and itching about the anus had hardly troubled her since.

Miss A. E., 20 years of age, had long been the victim of dysmenorrhœa, pain between her monthly periods, in the hypogastric region, and leucorrhœa; with general debility and anemia.

I found that the uterus was much anteflexed, but that by giving a flexible sound a curve forward, the usual size could be readily introduced. I purposely did nothing but elevate the anteflexed fundus and support it.

Although my patient was pale, thin and had frequent palpitations, was costive, full of pains, nervous, and unable to walk with ease, I avoided giving tonics, purgatives, anodynes, or nervines, by way of experiment. I *elevated* or *straightened* the uterus with a sound, using but the ordinary size, and applied a proper pessary, (one of Thomas') and made the patient wear it *right through the menstrual period*, which has occurred nine times since. The treatment extended over three months.

The patient had not for years been as free from pain as during the first period after treatment began, and each subsequent month was better than the last. The almost constant pain above the os pubis disappeared by degrees—she recovered appetite, strength, plumpness, and her rosy cheeks, and finally married in August last. I have within a few weeks received the pleasant intelligence, that, at some future day, I shall have to attend her for an obstetric case.

Finally I have notes of five cases of anteflexion in which the patients not being able to afford time or money to go through a regular course of treatment, I simply applied a pessary. Some of these have retained them for three or four months at a time, and have then come to assure me they were much benefitted.

Let us now state briefly the causes which may bring about constriction of the cervical canal and thereby cause dysmenorrhœa.

*Foremost* in the list is *flexion*, occurring at any part of the uterine canal, but most frequently at the *internal os uteri*—it may or may not be accompanied by version.

Anteflexion is more frequent than retroflexion, especially in the unmarried, it is much more likely to remain undiscovered. Anteflexion appears to be very prevalent in manufacturing cities, if Cohoes can be taken as an example.

*Congenital narrowness* of the os uteri *internum* or *externum*, or of the whole cervical canal—the latter condition being often associated with the presence of an infantile uterus.

*Congestion of the Cervix.*—When the cervical canal would be large enough under ordinary circumstances, it may become too small for the function it has to perform, in consequence of undue congestion, and hypertrophy of its lining membrane; or too increased flow of the blood from the cavity of the uterus.

*Chronic Congestion* of the *uterus itself*, generally associated with some amount of flexion.

There remain as causes, *Fibroids*, which compress or distort the cervical canal,—*small intra-uterine polypi* hanging down within the cervical canal and acting as a plug,—*Elongated* and often *flexed* cervix uteri, and *Contortion* of the cervical canal, due to irregular hypertrophy of the neck of the womb.

SELECTION OF CASES.

We now come to our second question, or the selection of those cases in which we may expect benefit from mechanical methods of treatment. Leaving out those purely due to congestion of the uterus, without change of shape, and which would probably be best treated by local depletion, we may expect benefit from mechanical modes of treatment, *in all cases* in which we can clearly diagnose either *flexion* or *contraction* of the uterine canal or *some impediment* to the egress of blood through it from a *polypus*, *fibroid* or some other adventitious growth, or any of the causes of *obstruction* before mentioned.

If we admit as a *fact* that the greater number of cases of ordinary dysmenorrhœa are due to *flexion* of the uterus, it follows that the correction of this flexion will relieve the majority of cases, and such I believe to be the truth. It must not be inferred from this, that I advocate carelessness of diagnosis.

I would not have every patient put on the bed of Procrustes, so to speak, and subjected without discrimination to dilatation and the use of the pessary—far from it! There are, no doubt, absolutely speaking, many cases of dysmenorrhœa altogether independent of flexion, and in which the treatment suited to that condition would be ridiculously inappropriate, if not positively hurtful—but it would lengthen this paper beyond all reasonable bounds to enter into their diagnosis. In every case we undertake to treat, we should make an exhaustive diagnosis—an object not generally to be obtained at one sitting, but as the result of repeated examinations. Besides the general acumen and delicacy of the "*tactus erudites*," the finger has to learn, so to speak, how to feel in any peculiar case, and it then often resolves problems that seemed hopelessly obscured at first. Moreover, the diagnosis being satisfactory before commencing treatment the surgeon should conscientiously weigh, in his own mind, whether the case is urgent enough to counterbalance the often serious objection to local treatment—especially in the virgin. While deprecating, as unjustifiable, the employment of local means when not imperatively called for, I yet believe that when dysmenorrhœa is a cause of continued impaired health, or of great pain, when it makes life cheerless, and without enjoyment, or causes the duties that devolve on the patient to become impossible, without the endurance of much suffering, *it then* is not only *justifiable* but *laudable* in the surgeon to interfere for the relief of the patient, even if she be a virgin. I do not hold that a maiden is *bound* to wait until the bloom has left her cheek, and the fire has departed from her eye, till she has become spiritless, and without hope in the future, and *then* and *then only* submit to treatment, which if employed months or years before, would have saved her to health and happiness.

Apart from the certain diagnosis to be arrived at by the patient examination, two subjective symptoms are of much importance, as pointing, *the one* to *obstruction* to the free passage of the menstrual fluid, *the other* to *anteflexion* of the uterus.

The first of these symptoms is intermittent pain at the menstrual period, and the second, pain in the inguinal region, generally confined to one side.

Had we these two symptoms in a given case, we would expect to find anteflexion tending to one side—that on which the pain was felt; and were we to find from bi-manual examination that such was the case, we would conclude that this anteflexion *obstructs* the free exit of the menstrual blood, *even* if we could pass a No. 8 catheter into the uterus without difficulty. Dr. Bennet and his school would hold that there cannot be any obstruction in such a case, but it appears possible, that having their minds fixed on the size of the tube, they have not considered sufficiently, that this tube may be bent on itself, thus offering resistance to the flow of menstrual fluid, yet yielding without the exertion of much force before a solid sound.

### TREATMENT.

With respect to the treatment of dysmenorrhœa it is not my intention to inflict on your patient ears, a description of the multifarious pessaries that have been invented, nor even to give my views on the relative merits of the different operative measures that have been employed, either to replace the uterus in its proper position, to dilate the cervix, or to extirpate foreign growths, such a work, on my part, would be one of supererogation, when addressing this learned assembly, many members of which are my seniors, and have, undoubtedly, more information and experience on the subject than myself. I shall, therefore, briefly state some of the modes of treatment I have used, and bring to your notice an operation for the relief of dysmenorrhœa, which is, as far as I know, comparatively new and unpracticed, and which may, therefore, be of interest to the society. I refer to the forcible and rapid dilitation of the cervix uteri, as proposed by Dr. John Ball, of Brooklyn.

The ordinary cases of dysmenorrhœa, and of other pains referable to the uterus, as they occur in office practice in Cohoes, are generally due to *anteflexion*, with or without narrowness of the cervical canal. These conditions are occasionally accompanied by a congested and tender state of the cervix and os uteri.

Two years ago, I used to commence the treatment of such cases by local depletion, practised by passing a narrow, sharp knife as far as the os internum, and incising down to the external os, to the depth of from one-eighth to one-quarter of an inch. The patient was put to bed, and a hot poultice applied over the vulva. Considerable depletion was thus obtained, the patient was kept in bed three days, and in one week afterwards I commenced dilatation by means of graduated sounds, and made the patient wear a pessary.

Latterly I have very seldom used the depletion, and find that the congested and irritable condition of the os and cervix disappears as the treatment by sound and pessary progresses.

Could you, in all cases, procure rest for your patient, it would be a great advantage, but practically this condition, if insisted on, interferes so much with your patients occupations or pleasures, that it deters her from profiting by the advantages of treatment, at least for some time, that is, until she becomes worse. Happily in cases of ordinary severity, and not very long standing, much relief is obtained, even when the patient continues her usual avocations.

The means I have used for both *dilatation* and *replacement* of the flexed uterus, are a series of metallic sounds of different sizes and degrees of curvatures. The patient comes to the office twice a week, and I introduce the sound, leaving it in the uterus about ten minutes. I generally use two sizes at every sitting, at first, one that can be passed without difficulty, and immediately on its removal the next size larger. If the uterus is flexed, as is generally the case, it is turned into the opposite position, by rotating the sound, and holding it thus, for ten or fifteen minutes. Then a proper pessary is put into the vagina, and retained continually. The pessary used is an ordinary *ring pessary* moulded into an *ovoid* shape for cases of *retroversion*, and into what Dr. Graily Hewitt calls "*cradle*," for cases of *anteversion*. For the latter the pessary of Dr. Thomas, likewise answers very well. All pessaries that have a stem and support outside the vagina displease most patients, who, unless the symptoms are very severe, are sure to discontinue their use. Nor are such disagreeable expedients necessary for all ordinary cases of dysmenorrhœa that are not of very long duration.

The *block tin rings* that can be found at Martin's in this city, are extremely convenient, and in a few moments can be bent into any shape one desires. I have never known them to cause irritation.

By means of this treatment, combined with tonics and purgatives, if indicated, *most* of the cases progress to the satisfaction both of physician and patient, and in three or four months are permanently relieved, at least to a great extent.

An anodyne containing Hyoscyamus, Indian Hemp and Chloric Ether, is generally proper for the first period or two after commencing treatment, and then generally becomes unnecessary.

If there is *lateral version*, one arm or side of the cradle pessary is made to project upwards and supports the uterus in its proper position. If there is *narrowness* of the cervical canal, special attention is paid to using graduated sounds until a No. 12 can be passed with ease.

In *strong* contrast, as the foregoing statements may appear, to those of many of the most eminent gynecologists, I, nevertheless, make them with confidence, based as they are on actual observation, and supported by the corroborative testimony of names as eminent as any in the profession, which give me encouragement in stating my own short experience. It is possible, that the *apparently irreconcilable* discrepancy in the opinions of these men of equal opportunities and acquirements, may be *explained* by supposing that *one class* includes only *intractable cases*, and the other *all cases* of dysmenorrhœa. In estimating the value of treatment, it is *no more* fair to base one's deductions only on cases that have resisted all treatment and have continued for a very long time in this disease, than in any chronic ailment. What would be thought of judging of the efficacy of a mode of treatment for Chronic Bronchitis or Rheumatism, if tested by those cases *only* which had proved themselves *inveterate*, and had resisted all other modes of medication. Would it not at once be said that to give the method a fair opportunity of manifesting its *comparative* value, it should be employed under the *same* circumstances as the treatment with which it is compared, and would not the opinion unavoidably force itself on an impartial mind that had these intractable cases been treated earlier, they might never have assumed their present unpromising character.

Whether the improvement is due to a correction of the flexion, or to distention of the canal of the cervix, (which partly or wholly obviates the effects of the flexion,) is not certain. I believe that in the majority of the cases it is a combination of both these effects, that brings about the good results which follow this plan of treatment. In this connection an important distinction has to be made between *version* and *flexion*. The *version* is, in all probability, very rarely cured, but the *flexion* being relieved, the obstruction to the flow of blood is removed. In other words, although the uterus is not reinstated in its normal position, the canal of the cervix is rendered straight in itself. It is *in* my opinion, the delay that occurs before attempting local mechanical treatment, which, (in many cases,) is the cause of its want of success when employed—the same is the case with respect to almost all methods of treatment in any disease whatever, in affording relief to which, as in most other things, " *Qui cito dat bis dat.*"

A few cases by way of example: Mrs. W., aged 24, married three years, no children, always had painful menstruation, and pains between periods increased by locomotion. Diagnosed anteflexed uterus with congested os. Used graduated sounds for two months, and a cradle pessary for three months: Patient felt relief from the first, and became pregnant while wearing the pessary. In due time she was delivered of a fine male child, has enjoyed excellent health since, and is now reaching the end of her second pregnancy.

Miss F., applied a year before her marriage for relief of her dysmenorrhœa, and constant bearing down pains, referable to the uterus. In this case marked constitutional symptoms were present, anemia, want of appetite and constipation. The cervical canal was very small and the uterus anteflexed. Same treatment as in last case, in conjunction with tonics, continued for six months, when she considered herself cured. Married six months later and had a fine boy.

Miss B., æt. 27, has had dysmenorrhœa for years, telling much on her general health. Treatment by graduated sounds and cradle pessary for three months. Relief almost immediate. Gained 14 lbs, weight in three months, and left, rejoicing.

Gentlemen, many such cases might be adduced with slight variations, but you will say, " what of those patients who do not progress favorably—whose case are either more severe to commence with, or less amenable to treatment ?

First of all we have the various *dilators* hitherto in use, which might be tried. There is Dr. Priestley's instrument, and those of Marion Sims, Mott, and many others, but it is doubtful if they present any real advantage over the series of graduated sounds. Their action is only limited, and if there is much induration of the tissues, they cannot be relied upon.

*Sponge tents* or *tents* of *Sea Tangle*, are much more reliable, but apart from their unpleasantness, and sometimes disagreeable effects, it will be found, that in cases of contraction that resist the treatment by the graduated sounds, their effect is not permanent.

*Intra-uterine stem pessaries* are more valuable, if retained without producing irritation, than either of the above means, *I believe*, but often cannot be worn by those who are employed in active avocations.

Dr. Schrœder, in Ziemssen's Encyclopœdia of Medicine, advocates the use of intra-uterine stems, with a vaginal bulb, both as repositors and permanent pessaries, for retaining the uterus in the proper position. This is done by packing the vagina with pellets of cotton, so placed against the vaginal bulb as to give the desired direction to the stem.

Dr. Schrœder's directions for introducing the stem contain a hint that will be often available—after introducing the stem as far as it goes without difficulty, (which is generally to the os internum,) he then, by pressing the cervix, by means of the bulb, backwards or forwards, makes it become straight, or in a line with the cavity of the anteflexed or retroflexed corpus—the stem then runs in with ease, and without bruising the uterine walls. By this manœuvre, the anteflexion or retroflexion has been converted into a *version* of the same kind, and this is reduced by pushing the bulb *backwards* or *forwards* ; when it can be retained in position by cotton in the vagina as before stated.

These means failing, we might insist on rest in bed, in a proper position, in conjunction with the intra-uterine stem pessary, but it is *that* very circumstance which disgusts a patient, who hardly feels sick enough to be confined *thus*, especially, when nothing very active is being done for her relief.

It is in such cases that an *operation* is demanded, and this brings us to the last subject of our essay.

What operations can we perform in such a case, if requested to do so by our patient ?

I will mention the operation of Koberle of Strasbourg, only because I am certain few of us will be tempted to repeat it. In a case of retroflexion he performed gastrotomy, and fixed the uterus to the abdominal wall by means of the broad ligament which he fastened to the outside wound. He cured the retroflexion, without killing the patient, which was a remarkable performance.

We have Marion Sims' operation for obstinate anteflexion, by slitting the posterior wall of the cervix, which I have performed once with a result satisfactory to my patient. This is, of course, applicable to nothing else than anteflexion, and is a formidable proceeding, as much hemorrhage is apt to occur.

We have also the *bi-lateral incision*, which has been, hitherto, the *dernier ressort* in these cases.

Whether performed according to the original plan of Sir J. Y.

Simpson, or according to the methods of Sims, Greenhalgh, Barnes or Coghlan, this operation essentially consists in incising both sides of the cervix, from the internal to the external os, and plugging the wound to prevent reunion of the cut edges.

All authorities on the subject allow that the operation is not without risks to life, and that the opening thus produced, invariably contracts after a certain time, and is apt to become almost as small as before. In fact, Hewitt proposes to use this operation in certain cases, as a first step towards subsequent treatment by intra-uterine stems, &c. Even Sims, who is a warm advocate, allaws both the objections I have stated.

With a view of furnishing a *prompt* and *decisive* means of treating cases of contracted or flexed cervix, which would be free from these objections, Dr. Ball, of Brooklyn, proposes to dilate the cervix *forcibly* and *rapidly* by means of a proper instrument, worked by a screw, and then to insert a large intra-uterine pessary, which is to be retained *in situ* until the uterus heals over it to the desired size, and in the requisite position.

Here is an instrument for the operation, and suitable intra-uterine pessaries which I had made a short time ago for a patient who desired me to operate. It has been used twice; once by myself, and once by our esteemed Vice-President, Dr. Featherstonhaugh.

Dr. Ball claims for his operation, the following advantages over the bi-lateral incision:

1. It is safer.*

2. It remedies *flexion* as well as *contraction* of the cervical canal, by causing an altered action in the nutrition of the cervix, and a certain amount of inflammation, which gives the uterus the needful stiffness, while by means of the stem it is made to heal in the desired position, which it retains.

3. The increase in size of the canal is not subject to be lost by cicatrization and consequent contraction.

4. It obviates the presence of a *cicatrix*, which is an objection

---

* In his pamphlet Dr. Ball says no bad symptoms have followed its employment. However, in a letter I received from the doctor within the last few days, though he reports many successful cases yet he mentions, also, one that proved fatal by peritonitis, brought on, the doctor believes, by the imprudences committed by the patient.

during parturition, and it does not endanger the life of the fœtus by miscarriage from destruction of the structure of the cervix, the natural guardian of pregnancy.

The manner of performing the operation will be fully described in the report of the two following cases.

Miss A. N., 24 years of age, began to menstruate early, and had much pain from the commencement, which diminished somewhat as she became more developed, until she entered a knitting mill, where she had for several years, to stand from six A. M. to six P. M., with the exception of three-quarters of an hour for dinner. For the last few years her work has been sitting, bent forward. Most of the work had to be accomplished with the left hand, with which arm all the lifting had to be done.

During the last two years her symptoms have much increased in severity.

A few hours after the flow had appeared in trifling proportions, the pains commenced. They began in the back, coming round the hips to the hypogastric region, and were of a pressing down or expulsive nature. The patient compares them to forcibly pressing the blood down into a sore finger. The result of these pains would be a more free discharge, after which a little ease was felt, for a short period, followed by the same pain, resulting in the same consequences.

After the first day the pain would diminish, until the third, when the same train of symptoms would return, with the difference that the uterus felt "*sore*," and the pains would not intermit or remit as completely as on the first day.

The patient had been troubled for about three years with vesical tenesmus, which was increased at the menstrual period, and, for about the same extent of time, has had much sympathetic pain in the epigastrium, often severe enough to make her quit work and take to bed. She also had the characteristic pain in the left inguinal region which was never felt on the opposite side. Her appetite was capricious and often absent, and she lost strength and was subject to sick headache of a neuralgic character, and to deranged stomach.

In May last, the patient came under treatment. Guided to the true nature of the case by the pain in the left inguinal region, I made an examination, and found the uterus presented a typical case of *antero lateral flexion* and *version;* it being bent on itself quite acutely, and the fundus laying forwards in the left inguinal region, where it could be distinctly pinched, so to speak, between the finger in the vagina and the hand on the abdomen. The os gave the impression of being inserted into the left side of the vagina, instead of its median line, and a sound to be introduced had to be directed towards that side.

A Sims' silver probe, representing a No. 4 catheter, bent almost to a right angle, and introduced with the concavity to the left side, was all that could be made to pass the os internum, and that with great difficulty. The uterus itself was very small, almost infantile in size, and a sound which completely reached the fundus entered barely one inch and six-eighths.

There was therefore a small uterus with a narrow canal and *flexion* and *version antero laterally.*

The treatment adopted consisted of dilatation with the graduated metalic sounds, and reposition by their means, as before described, and the insertion into the vagina of a block tin pessary, of which one arm projected upwards considerably more than the other.

The patient improved sensibly and her menstrual periods were much less severe, especially if she were careful to have the dilatation practised regularly twice a week; but she neglected to do so and found her old troubles returned.

Having asked me if some more permanent relief could be obtained, I showed her one of Dr. Ball's pamphlets, and told her to take it home to her mother.

I represented the operation to her, gentlemen, as I do to you, for what it is worth; telling her I knew nothing whatever about it, but what was in that pamphlet; and that while I was willing to try it, in her case, if she desired, I must leave the entire matter to her own judgment.

The result was she decided on having the operation performed. I accordingly had the instrument you see and suitable intra-uterine pessaries made by Tiemann.

Dr. Ball advises to bring the patient into a state of profound anasthesia, and then to open the os uteri to a sufficient amount to introduced the dilator, by first using the largest metal bougie that will enter with ease, and following with larger ones in rapid succession until the desired size is attained. In this case, my patient having a mitral systolic murmur, I wished to employ as little of the anasthetic as possible, and the uterus being very tolerant of manipulation, I introduced graduated sounds every day for a week and succeeded on the eve of the operation in placing the dilator *in situ.*

The patient having taken a purgative the day before, I operated on January 12th, assisted by Drs. Dunlap and Featherstonhaugh.

To abridge the period of anasthesia before administering the ether, the patient was placed in position on her back, the hips well out over the edge of the bed, and her feet resting on chairs; a three bladed self-retaining speculum was then introduced, the os uteri transfixed by a tenaculum and pulled down and the dilator placed in position.

Ether was now administered, and as soon as the patient was under its effects the screw was turned. The dilator had been opened to the size of half an inch, when to the great disappointment of all concerned, the central rivet or pivot broke, and the operation had to be left incomplete.

The patient not at all discourged by this failure said she would try it again, but as her menstrual period would soon occur, it was deemed prudent to await its completion.

She commenced menstruating within seven days, and the period was less painful than usual, but only to a trifling degree.

The patient was kept most of the time in bed, and had no other trouble than a little of her usual vesical tenesmus; however this confinement, and the delays that took place, somewhat diminished her general good condition.

January 28th having been appointed as the day, she insisted upon the operation being performed in spite of her having a sick headache.

Having found she could stand ether well, she was put completely under its influence, and assisted by Drs. Dunlap, Featherstonhaugh and Witbeck, I proceeded as before. The uterine canal was dilated in all directions to about the three-seventh of an inch, but not as much as I would have desired. It was very difficult to distend, and our rivet again broke before I was fully satisfied. I cannot help, in this place, criticising manufacturers of so much experience, for putting so small a rivet in a position in which it has to bear the whole distending force. However, we could introduce this pessary which was done.

It was kept in place by tapes passed through this ring and secured by adhesive plaster on the back and abdomen—they were made quite tight. The patient was then put to bed, given ice to control vomiting that was present, and later a dose of morphia.

The constitutional disturbance that followed this operation was more than after the first, there being quick pulse, furred tongue, and pyrexia, but it all subsided in two or three days.

I now discovered that the pessary being put on a screw, and not fixed firmly on a rod, as should have been done, it allowed the uterus to fall towards the left side. To remedy this cost me considerable trouble, as the gutta percha part of the pessary was wholly within the vagina, but I finally succeeded.

I mention these apparently trifling incidents that any who may desire to try the operation, may profit by my annoyances, and take care that all the appliances are perfect before commencing the operation. Another little practical point is this—the tenaculum should be passed from within the os uteri outwards, through its wall, its point does not then interfere with the action of the dilator. It is important likewise to firmly transfix the wall of the cervix, otherwise the tenaculum will tear out, causing an ugly laceration.

The patient did well, and I intended to remove the pessary at the end of the week, but menstruation having commenced, I thought I had better leave it in till that process was terminated in order to let those changes which occur during the period take place while it was *in situ*.

The most gratifying result was that menstruation took place almost without pain, and what trifling discomfort there was present, was of the same character as that she had the days before, and attributed to the presence of the pessary in the uterus.

I look upon this circumstance as satisfactory, no matter what may be the final result of the operation, for it proves to my mind that the pain was due to mechanical obstruction to the free discharge of the menstrual blood, for here was the uterus, yet in an inflammed, or at least irritated condition, that could not be touched without causing pain, and which, nevertheless, was menstruating without suffering, while the same organ, when it had not a particle of inflammation or irritation, when it could be handled, probed and dilated without causing even passing irritation, could not then perform its monthly function without causing intense distress.

I removed the pessary two days after menstruation ceased, without producing pain, and two days later the patient began to sit up. As a precaution, I introduced her pessary, for the vagina had been considerably stretched by the upward pressure of the strings on the pessary inserted into the uterus.

My patient was a little *run down* by her prolonged confinement in bed, but apart from a few nervous symptoms, she has progressed well, and is now able to take a long walk, and gains strength from day to day. A few days ago, for the sake of information, I passed a No. 10 sound, which glided in of its own weight as it would into a capacious male urethra, and without producing any but trifling distress.

The following are the notes taken on his case by Dr. Featherstonbaugh, and kindly put at my disposal for this occasion.

The doctor commenced treating Mrs. B., on January 1st. She was suffering from profuse diarrhea, having five to fifteen passages a day. She had been afflicted with this complaint for five or six years, accompanied with constant pain in the back, rectum and inferior extremities. Had vomited almost every day for two or three years, and was unable for two on three weeks in every month to walk but short distances. Menstruation was very painful, and leucorrhœa existed. For about a week the patient was treated with Tannate of Bismuth and Morphia, without modifying the symptoms.

January 10th—*Examination.*—Large œdematous cervix—granular erosions, and easily bleeding surface. Tannin, Glycerine, Tincture of opium and extract of Balsam of Pinus Canadensis, were applied locally to January 15th, on which day dilatation of the cervix by sponge tents was commenced.

January 18th.—Having examined with a sound, an acute retroflexion was discovered, applied a retroflexion pessary.

Used several pessaries up to January 30th with about the same result, which was *nil.*

February 2d.—Dilated under chloroform with instrument according to the method of Dr. Ball, and introduced this large hollow stem pessary, which was retained in the uterus by tapes as prescribed in last case.

No constitutional symptoms whatever followed the operation.

February 4th.—Constipation.

February 10th.—Menstruating through the hollow pessary without pain—has not vomited since the operation.

February 13th.—Menses ceased.

February 17th.—Withdrew pessary—condition of patient good.

February 20th.—Sound No. 16 passes into the uterus and shows its position to be normal.

March 6th.—Simpson's sound passes easily into the uterus, with the usual curvature. Patient says she menstruated without pain on March 3d, and is still menstruating. Has one or two normal passages a day, does not vomit, and expresses herself as very well.

### CONCLUSION.

In conclusion let me add, that in his pamphlet Dr. Ball mentions that a somewhat similar idea has been enunciated by Dr. J. Protheroe Smith, of London, whose method, however, was much less complete, and extended over much time, and who did not use a pessary. After writing his paper, Dr. Ball's attention was called to an article translated from the German, of Dr. Ellinger, of Stuttgart, and published in the New York Medical Journal, containing a variety of experience similar to his own, with the exception of the use of the pessary. Dr. Ellinger's method is mentioned in Ziemssen's Encyclopœdia.

Dr. Ellinger employs, for rapid dilatation, a sort of modified polypus forceps, which are introduced into the narrowest cervix without preliminary dilatation. In case any part of the canal offers any resistance to the progress of the instrument, by separating the arms of the instrument for a few moments, the stricture above is found to yield, so that in this way a cervix which offers great difficulties to the introduction of the sound, allows the dilatorium to pass with facility. The pain is said to be no greater than that induced by the introduction of the ordinary Simpson sound. In general, dilatation was performed upon office patients who were allowed to go about their business.

Dr. Ellinger recommends extemporized dilatation :

1. In stricture of the cervical canal.
2. In Stenosis due to flexions.
3. Metrorrhagia in a flabby, swollen uterus, but without new growths.
4. Retained catarrhal secretions.
5. For exploration of uterine cavity.
6. Replacement of a flexed uterus.
7. Sterility.

Finally, Dr. Ellinger declares that he has *never* had reason to regret the rapid dilatation, and urges it where dilatation is justifiable at all, to the exclusion of all other methods.

With respect to *counterindications* to the operation, Dr. Ball says he would recommend it where any other surgical or mechanical means would be considered advisable.

He would not interfere, however, in any case where there was acute inflammation of any part of the organ, yet, in the body of his pamphlet Dr. Ball says : " I have operated upon patients who had suffered for years from chronic eudo-cervicitis, and when the most gentle touch of the finger would cause excessive pain, when, in a few days the sensibility would all be gone, sometimes even before the pessary was removed."

Should this dilator prove to possess advantages over those hitherto introduced, I propose to have a slighter one made with the natural curve of the uterine canal, to use in the office, for patients who prefer their treatment to extend over a longer period, than to be put to the trouble and confinement incident to an operation.

I also think that the edges might with advantage be made more rounded, like those of an ordinary sound.

Whether this operation will merit the claims put forth by its originator remains for the future to demonstrate.

In the two cases in which it has been employed in Cohoes, the result is, so far, favorable, but it is only the combined experience, derived from many operations, that can decide whether it is destined, like so many other innovations, to sink into oblivion, or to be maintained as a real step forward in gynécology, a department of our science, which born of this century, has, notwithstanding its youth, been second to none in the rapidity of its march towards excellence, and which has done *so much* to alleviate the suffering of that interesting portion of our species to which we all owe so much, both as men and physicians.

